# Could Selected Adipokines/Cytokines Serve as Markers of Adipose Tissue Dysfunction?

**DOI:** 10.3390/ijms252413744

**Published:** 2024-12-23

**Authors:** Lucyna Ostrowska, Joanna Smarkusz-Zarzecka, Beata Zyśk, Karolina Orywal, Barbara Mroczko, Urszula Cwalina

**Affiliations:** 1 Department of Dietetics and Clinical Nutrition, Medical University of Bialystok, ul. Mieszka I 4B, 15-054 Bialystok, Poland; lucyna.ostrowska@umb.edu.pl (L.O.);; 2 Department of Biochemical Diagnostics, Medical University of Bialystok, ul. Waszyngtona 15A, 15-269 Bialystok, Polandbarbara.mroczko@umb.edu.pl (B.M.); 3 Department of Biostatistics and Medical Informatics, Medical University of Bialystok, ul. Szpitalna 37, 15-295 Bialystok, Poland; urszula.cwalina@umb.edu.pl

**Keywords:** adipose tissue, inflammation, obesity, adipokines, cytokines

## Abstract

Elevated levels of pro-inflammatory adipokines and cytokines increase the risk of developing metabolic disorders and diseases. The aim of this study was to conduct a comparative analysis of selected adipokines/cytokines in the blood serum of adults with obesity and normal body weight. The study also evaluated the correlation of these adipokines/cytokines with selected biochemical blood parameters. The study included 46 individuals with first- and second-degree obesity and 35 individuals with normal body weight. The participants underwent nutritional status assessments, biochemical tests, and evaluations of adipokine and cytokine concentrations in blood serum. The study found higher median CRP concentrations in women with obesity than in those with normal weight. This increase was statistically significant. The results also showed significantly higher IL-6 levels in the obesity group compared to the control group in both women and men. Resistin and MMP-2 were significantly different between women with obesity and women with normal body weight. Multiple regression results indicated that higher total fat content was significantly associated with higher serum CRP and IL-6 levels and lower adiponectin levels. Interleukin 6 was the strongest predictor of adipose tissue dysfunction in both women and men. Potential markers in women could also include resistin and MMP-2. The findings suggest that gender significantly influences the regulation of inflammatory factors.

## 1. Introduction

Over the past several years, there has been a significant increase in the global prevalence of obesity. The prevalence of obesity worldwide nearly tripled from 1975 to 2016. According to data from the World Health Organization (WHO), in 2016, approximately 39% of adults were overweight, and 13% were obese [1]. A similar trend has been observed in Poland. Reports from the World Obesity Federation (WOF) indicate that the percentage of overweight individuals increased from 37.6% to 46.8% among men, and from 26.5% to 32.2% among women, between 1996 and 2019. During the same period, the prevalence of obesity increased from 10.3% to 20.1% among men, and from 12.4% to 18.1% among women [2].

Obesity leads to the development of over 200 complications, including non-alcoholic fatty liver disease (NAFLD), type 2 diabetes, cardiovascular diseases, and urogenital disorders [3,4,5]. A promising direction in research for diagnosing obesity-related complications is the evaluation of adipokine and cytokine levels in serum. This is due to the significant role of chronic low-grade inflammation in the pathogenesis of obesity. These measurements could be performed as part of routine diagnostics during periodic biochemical blood tests.

In obesity, there is an increased influx of macrophages into adipose tissue, which depends on the degree of obesity. This process leads to the development of inflammation and insulin resistance. At the same time, weight reduction contributes to a decrease in inflammation. Additionally, these immune cells exhibit different phenotypes and, as a result, perform distinct functions depending on the patient’s nutritional status. In individuals with obesity, M1 macrophages predominate. Their activation increases the secretion of pro-inflammatory cytokines, such as tumor necrosis factor-alpha (TNF-α) and interleukins IL-6, IL-12, and IL-23. At the same time, it decreases the synthesis of the anti-inflammatory interleukin IL-10. In contrast, the adipose tissue of individuals with normal body weight contains M2 macrophages. These macrophages help to prevent inflammation by secreting higher amounts of the anti-inflammatory interleukin IL-10 and lower levels of the pro-inflammatory interleukins IL-12 and IL-23 [5,6,7].

Obesity contributes to the increased activity of adipocytes and macrophages. Stimulated cells secrete greater amounts of adipokines and pro-inflammatory cytokines, which may contribute to the development of other metabolic disorders associated with obesity. However, the effects of these cytokines depend on their type. Resistin induces insulin resistance and glucose intolerance, and also contributes to the development of cardiovascular disease. Interleukin 1β negatively affects carbohydrate metabolism. In contrast, the matrix metalloproteinases MMP-2 and MMP-9 exhibit atherosclerotic activity. Additionally, MMP-2 may have a profibrotic function, playing an important role in the development of liver fibrosis. On the other hand, adiponectin and IL-6 concentrations are positively correlated with tissue insulin sensitivity. Notably, adiponectin concentrations are reduced in individuals with obesity-related conditions, as shown in studies conducted to date [6,8,9,10,11,12,13,14].

We conducted this study to perform a comparative analysis of the levels of selected adipokines and cytokines in the serum of adults with obesity compared to individuals with normal body weight. Additionally, we evaluated the correlation between these adipokines and cytokines in serum, and selected biochemical blood parameters.

## 2. Results

### 2.1. Anthropometric Features

Descriptive statistics were performed. The characteristics of the study groups in terms of anthropometric features are presented in Table 1. Statistically significant differences in both women and men were observed in almost all of the studied parameters (with the exception of the VAT/SAT ratio in the male group, and height in both groups). Additionally, women in the study group differed significantly in age compared to women in the control group. However, in the case of men, the differences in this characteristic were not statistically significant.

### 2.2. Comparison of Inflammatory Marker Levels in Relation to Body Weight and Gender

In the group of women with obesity compared to those with normal body weight, significantly higher serum levels were found for IL-6, MMP-2, and resistin. In the case of men, a statistically significant difference was observed only in the serum level of IL-6. The discussed results are presented in Table 2.

### 2.3. Evaluation of the Correlation Between Serum Cytokines and Adipokines and Selected Anthropometric Parameters in All Women and Men Studied

An analysis of the correlations between cytokines and adipokines in the blood serum and selected anthropometric parameters of all the studied women was performed. It was found that only IL-6 showed a statistically significant correlation with nearly all the analyzed parameters (excluding WHR). For TNF-α, a statistically significant correlation was observed only for WHR. For MMP-2, statistically significant correlations were observed with BMI, waist and hip circumference, and fat content (in kg and %), as well as subcutaneous fat content (cm^2^). In contrast, resistin showed statistically significant positive correlations with hip circumference and fat content (kg). As for IL-1β, statistically significant correlations were observed with waist circumference, fat content (%), and visceral and subcutaneous fat content (cm^2^). For adiponectin, MMP-9, and IL-23, no statistically significant correlations were found with the anthropometric parameters and body composition of the studied women. The discussed data are presented in Table 3.

Similar analyses were performed in the group of all the studied men. Only IL-6 showed a statistically significant correlation with waist circumference, hip circumference, and WHR (waist-to-hip ratio). No statistically significant correlations were found for the other analyzed adipokines and cytokines. The discussed data are presented in Table 4.

### 2.4. Assessment of Selected Biochemical Parameters

Next, an assessment of selected biochemical serum parameters was conducted for all the participants. Women with obesity, compared to those with normal body weight, showed statistically significant differences in fasting serum levels of creatinine, uric acid, and eGFR, as well as HDL and LDL cholesterol fractions, triglycerides, ALT, GGTP, ALP, CRP, and insulin. In men with obesity, compared to those with normal body weight, statistically significant differences were observed in fasting serum levels of insulin, uric acid, LDL cholesterol, triglycerides, ALT, GGTP, and glucose levels after two hours in an OGTT. The discussed results are presented in Table 5.

### 2.5. Correlation Analysis Between CRP Levels and Fat Tissue Parameters

Next, we analyzed the correlation between CRP levels in the blood serum and total body fat content (%), VAT and SAT areas in the cross-sectional abdominal plane (cm^2^), and VAT/SAT ratio. Statistically significant correlations were observed only in women. The results are presented in Table 6.

### 2.6. Evaluation of Adipokines and Cytokines as Markers of Inflammation

Next, a multivariate analysis was conducted in the group of women, with multiple regression models performed to assess whether adipokines and cytokines could serve as markers of adipose tissue inflammation. It was observed that a higher fat content was significantly associated with increased serum CRP levels, higher IL-6 levels, and lower serum adiponectin levels. Additionally, an increased VAT area in the cross-sectional abdominal plane showed a significant correlation with higher CRP levels. Furthermore, an increased SAT area in the cross-sectional abdominal plane was significantly associated with higher serum CRP levels. No significant correlations were observed for VAT/SAT ratio. The discussed results are presented in Table 7.

A multiple regression analysis was also conducted in the group of men, but no significant correlations were observed. The results are presented in Table 8.

### 2.7. Analysis of Adipokines and Cytokines in Relation to Selected Biochemical Parameters

Next, a correlation analysis was performed between serum cytokines and adipokines and selected biochemical parameters in all the studied women. A statistically significant positive correlation was observed between adiponectin levels and fasting serum HDL cholesterol levels, as well as a negative correlation with fasting glucose and insulin levels. A statistically significant negative correlation was observed between TNF-α levels and CRP, as well as with glucose after 1 h in an OGTT. The cytokine that showed the most statistically significant correlations was IL-6, with positive correlations observed with levels of uric acid, triglycerides, GGTP, ALP, CRP, and fasting insulin. A statistically significant positive correlation was demonstrated between MMP-2 and HDL cholesterol levels. For MMP-9, a statistically significant positive correlation was observed with total cholesterol, LDL cholesterol, triglycerides, and CRP levels. For resistin, a statistically significant positive correlation was observed with fasting insulin. IL-1β showed a statistically significant negative correlation with LDL and fasting insulin. For IL-23, a statistically significant negative correlation was observed with alkaline phosphatase and CRP levels, and a positive correlation with fasting glucose level. The discussed data are presented in Table 9.

Similar analyses were performed in the group of all the studied men. A statistically significant negative correlation was observed between ALT levels and TNF-α levels. IL-6 showed a statistically significant negative correlation with urea levels. A statistically significant positive correlation was demonstrated between MMP-9 and bilirubin concentration. A statistically significant negative correlation was demonstrated between IL-23 and glucose concentration after 2 h in an OGTT. For MMP-2, resistin, and IL-1β, no statistically significant correlations were observed. The discussed data are presented in Table 10.

## 3. Discussion

The global prevalence of obesity has increased among adults and children. At the same time, awareness within the medical and scientific communities regarding this disease and its complications has grown. These factors have led to significant advancements in obesity treatment. However, a major challenge remains in the accurate and effective diagnosis of this condition. Metabolic obesity can occur not only in individuals with increased body weight, but also in those with normal body weight. The concept of metabolic obesity was created to define people who have a normal body mass index (BMI) but who, due to the accumulation of visceral fat, are at increased risk of metabolic disorders [13]. This is known as MONW syndrome (metabolic obesity with normal weight). People with MONW syndrome, despite having a normal BMI, are characterized by insulin resistance, which may contribute to the development of other diseases, including diabetes and cardiovascular diseases [15]. Therefore, it is crucial to explore new diagnostic methods and markers for this disease and its complications. These methods should be widely available and cost-effective, so they can be used in daily clinical practice and serve as part of screening programs.

A promising research direction appears to be the analysis of adipokines/cytokines secreted by adipose tissue as potential markers of its inflammatory state, which can lead to both local and systemic consequences. Local inflammation of adipose tissue disrupts the normal differentiation of adipocytes and insulin signaling. It also leads to the development of chronic, low-grade systemic inflammation, which plays a crucial role in the pathogenesis of obesity. The dysfunction of adipose tissue cells leads to changes in the profile of adipokine and cytokine secretion toward a pro-inflammatory state. Increased expression of pro-inflammatory adipokines and cytokines, coupled with decreased secretion of anti-inflammatory adipokines/cytokines, adversely affects peripheral tissues. In our study, we analyzed changes in the levels of selected inflammatory parameters in individuals with obesity compared to those with normal body weight.

### 3.1. Comparison of Inflammatory Marker Levels in Relation to Body Weight and Gender

Our study found significantly higher levels of IL-6 in the obesity group compared to the control group (normal body weight) in both women and men. Similar results were obtained in a study by El-Mikkawy [16]. In a study by Popko et al., significant differences in IL-6 levels were also observed between the obesity group and the normal body weight group, but these significant differences were only found in women (*p* = 0.001) [17]. In a study by Sindhu et al., subcutaneous fat tissue biopsies were performed, and the expression of IL-6 and IL-6 receptor (IL-6R) were assessed. Higher expression of IL-6 and IL-6R were found in the adipose tissue of individuals with obesity compared to those with normal body weight or overweight [18]. Our study also revealed a significant positive correlation between the levels of this cytokine and nearly all the assessed anthropometric parameters, indicators (excluding WHR), and body composition components in women. In the group of men, such correlations were observed for waist circumference, hip circumference, and WHR. The study by Sindhu et al. found that elevated IL-6 expression was positively correlated with BMI (r = 0.80, *p* < 0.0001) and percentage of body fat (r = 0.69, *p* = 0.003) [18]. In a study by Valmorbida et al., a relationship (assessed using multiple regression) was observed between IL-6 levels and waist circumference, as well as BMI, in a group of women. In a group of men, a relationship was found between IL-6 levels and waist circumference. Additionally, correlations were observed between IL-6 levels and the WHtR index, as well as between IL-6 levels and BMI [19]. In contrast to the results obtained in our study and those of other researchers, Mendez-Garcia et al. reported a 2.5-fold significant decrease in serum IL-6 levels in individuals with overweight and obesity compared to a control group with normal body weight. Additionally, serum IL-6 showed significant negative correlations with BMI (r = −0.39, *p* < 0.0001) and waist circumference (r = −0.42, *p* < 0.001) [20]. Our study shows that IL-6 may be an important cytokine and marker of inflammation in people with obesity, because its concentration is significantly increased in the case of obesity. However, more research is needed to draw clear conclusions.

In this study, matrix metalloproteinase 2 and resistin were found to be significantly different between the group of women with obesity and the group of women with normal body weight. In the case of men, no similar significant differences were observed. The results of a study by Al-Mahdi et al. showed higher in women with obesity and overweight compared to women with normal body weight [21]. In contrast to our study, Valmorbida et al. did not observe any correlations of IL-1β levels with BMI, waist-to-hip ratio, fat content (in kg and %), and subcutaneous fat content (cm^2^) in a group of women. However, in men, a relationship was found between IL-1β levels and waist circumference. Additionally, correlations were noted between IL-1β levels and BMI [19]. The findings of Nieva-Vazquez et al., similar to those of this study, demonstrated a correlation between increased resistin levels and a higher degree of obesity [22]. The data obtained suggest that there may be a difference in the concentration of pro-inflammatory cytokines between individuals with different body weights. Additionally, these differences may result from the distribution of adipose tissue. However, the limited number of studies and conflicting results prevent clear conclusions, highlighting the need for more research. An important aspect of this is the analysis of adipose tissue distribution.

In our study, no differences were observed in the levels of IL-23 and MMP-9 between the study and control groups, for both women and men. However, TNF-α was statistically significantly negatively correlated with WHR in the group of women. Popko et al. found that TNF-α levels were significantly higher in individuals with obesity (*p* < 0.001) [17]. In a study by Fiorotti et al., changes in the levels of selected adipokines and cytokines were assessed in patients before and after Roux-en-Y gastric bypass. Post-operatively, there was a significant reduction in body weight (−26.8%), body fat mass (−50%), waist circumference (−18%), and waist-to-height ratio (−22%), which was associated with changes in the adipokine and cytokine profile. Serum adiponectin levels increased (+107%), while resistin (−12.2%), TNF-α (−35%), and PAI-1 (−11.1%) levels decreased [23]. In contrast, Grzechocińska et al. found a positive correlation between MMP-9 levels and BMI, noting that MMP-9 levels were significantly higher in women with obesity compared to women with a normal BMI [24]. Studies on cytokines in recent years are scarce. However, our results confirm previous assumptions. People with obesity-related diseases show increased inflammation and elevated production of pro-inflammatory cytokines, which correlate with higher body fat levels. As a result, there are elevated serum concentrations of these cytokines [25,26].

In our study, no differences in adiponectin levels were observed between the study group and the control group in both women and men. Multivariate regression analysis showed that levels of this adipokine decreased with an increase in total body fat content (%). Such a relationship was observed only in the group of women. Other researchers have not evaluated adiponectin for its potential use as a marker of adipose tissue dysfunction. It is worth conducting more studies to clearly determine whether adiponectin concentration is dependent on body mass. The distribution of adipose tissue in relation to adiponectin concentration should also be taken into account, because the type of adipose tissue may have a particularly important effect on adiponectin concentration.

### 3.2. Evaluation of CRP Levels in Relation to Body Weight, Body Fat Content and Distribution, and Gender

In our study, we found higher median CRP concentrations in women and men with obesity than in those with normal weight. Furthermore, in women, serum CRP levels were significantly correlated with adipose tissue parameters. No analogous significant correlations were observed in men. Similar results were obtained from a systematic review and meta-analysis (including 51 studies) by Choi et al., which also found that obesity was associated with higher CRP levels, with a stronger association in women. Additionally, the review observed a stronger correlation between CRP levels and obesity among Europeans and Americans compared to an Asian population [27]. In contrast, another systematic review (including 11 studies) demonstrated that the use of a low-energy diet in individuals with obesity was associated with lower CRP levels [28]. Our study is one of the few to assess CRP levels in people with obesity and compare them to those in people with normal weight. The observed differences between these groups may provide important insights into the severity of inflammation in individuals with obesity. Therefore, it is worth further investigating this issue and designing additional studies focused on this inflammatory marker.

### 3.3. Analysis of the Effects of Adipokines and Cytokines on Carbohydrate Metabolism

Our study found that women and men with obesity had higher median fasting glucose and insulin levels compared to women and men with normal body weight. This study explored the correlations between changes in serum adipokine and cytokine levels, and parameters of carbohydrate metabolism and insulin regulation. It was observed that with an increase in resistin levels in women, fasting insulin levels significantly increased. Similarly, Nieva-Vazquez et al. found a significant correlation (*p* < 0.001) between higher resistin levels, greater obesity, and decreased insulin sensitivity (IS). [22]. Su et al. demonstrated that in individuals with obesity and type 2 diabetes, resistin levels were positively correlated with insulin resistance in those with hyperresistinemia, but not in those with normal resistin levels [29]. In a study by Bastard et al., compared to a group with normal body weight, patients with obesity without diabetes and those with obesity and diabetes were characterized by greater insulin resistance and elevated IL-6 levels, similarly to our study [30]. In a study by Zak et al., it was demonstrated that patients with type 2 diabetes exhibited increased levels of IL-6 and IL-1β, which is also consistent with our results [31]. Additionally, in this study, a correlation was observed between higher adiponectin levels and lower fasting glucose and insulin levels. A multivariate regression analysis conducted by Muratsu et al. demonstrated an association between older age, lower BMI, higher adiponectin levels, and a higher HOMA-IR index (assessing the likelihood of insulin resistance) [32]. In a study by Diwan et al., adiponectin levels were significantly higher in individuals with diabetes compared to those without, regardless of gender (*p* ≤ 0.04 in men, *p* ≤ 0.02 in women) [33]. In our study, we found that in men, IL-23 was significantly negatively correlated with glucose levels after 2 h in an OGTT. However, Roohi et al. reported that serum IL-23 did not show any significant association with type 2 diabetes [34]. Our findings suggest that cytokines secreted as a result of increased inflammation in adipose tissue in individuals with obesity may significantly influence the development of insulin resistance. This aligns with previous hypotheses. Although there are still insufficient studies to fully evaluate the effects of IL-6, IL-23, and resistin on tissue insulin sensitivity, it can be assumed that they play a significant role in this mechanism. These conclusions are based on the defense mechanisms of increased insulin secretion and the resulting elevated serum glucose levels.

### 3.4. Analysis of the Effects of Adipokines and Cytokines on Liver Function

In this study, men with obesity exhibited higher levels of ALT, GGTP, and TG compared to men with a normal body weight. Similarly, women with excess body weight showed analogous differences in these parameters, with the addition of elevated ALP levels. In another study of ours, which analyzed a broad profile of serum adipokines and cytokines in patients with metabolic dysfunction-associated steatotic liver disease (MASLD) (assessed by elastography and other diagnostic criteria), IL-6 was identified as the strongest predictor of this disease, regardless of gender [35], a finding that is also supported by other researchers [36].

### 3.5. Analysis of the Influence of Adipokines and Cytokines on Lipid Profile

Although the primary cause of hypertriglyceridemia is genetic factors, excessive adipose tissue can lead to increased production of pro-inflammatory cytokines, resulting in elevated triglyceride levels and liver enzymes in the serum. Excessive adipose tissue, particularly when located around organs, stimulates an intensified inflammatory state, which disrupts lipid metabolism and may contribute to the development of serious cardiovascular diseases. Additionally, conditions such as non-alcoholic steatohepatitis may develop, leading to significant metabolic disorders. Research by Hernandez-Anzaldo et al., conducted on mice, demonstrated that MMP-9 at least partially modulates cholesterol metabolism. They found that dysregulation of MMP activity could lead to metabolic disorders, potentially contributing to the development of atherosclerosis and coronary artery disease [37]. Our results suggest that MMP-9 may have an impact on the regulation of the body’s lipid metabolism. As a result of the development of obesity-related disease, the function of the MMP system is disrupted, resulting in the disruption of lipid balance. Assessment of MMP levels can be useful in evaluating the cause of lipid disorders and cardiovascular disease. Additionally, it may be a new therapeutic target for lipid disorders in humans, particularly with obesity-related disease.

### 3.6. Analysis of the Effects of Adipokines and Cytokines on the Genitourinary System

Our study shows that cytokines produced by adipose tissue as a result of the development of inflammation in the course of obesity disease may have an impact on the development of impaired kidney function. A study by Navaneethan et al., evaluating the relationship between obesity and kidney function indices in patients with CKD, showed that obesity worsens the condition of patients. It was noted that excessive accumulation of adipose tissue increases protein excretion and biochemical indices, which reflect kidney function. Renal function indices were correlated with an increase in pro-inflammatory factors, including IL-6 [38]. However, more studies are needed to evaluate this correlation, as this may be a new target for the development of renal disease therapies, especially in people with obesity.

### 3.7. Limitations of the Study

A limitation of our study is the relatively small number of participants. This constraint primarily stems from the high cost of the reagents required for analyzing the selected pro-inflammatory cytokines and adipokines. Moreover, this study focused on cases of simple, uncomplicated obesity, with strict inclusion and exclusion criteria applied when selecting participants. While this approach significantly reduced the number of eligible individuals, it also ensured a carefully selected, highly representative study group, which can be viewed as a strength of the research. Moreover, our study included patients of varying ages (age range 29–46 years). In awareness of this limitation, we performed statistical calculations (multiple regression models) for the selected adipokines and cytokines from the study. In these models, a statistically significant relationship between BMI and the dependent variables was found, while no statistically significant relationship between age and the dependent variables was observed. Since this was a pilot study conducted on a small group of patients, it allowed us to determine that future analyses should focus on more precise patient selection regarding age and sex, to ensure that the groups are as comparable as possible. We believe that conducting further studies on larger populations could provide deeper insights into the mechanisms underlying the development of metabolic disorders linked to the concentrations of pro-inflammatory cytokines in the blood of obese patients. These findings could potentially be translated into practical applications in everyday clinical practice.

## 4. Materials and Methods

An observational study was conducted, with protocols approved by the Bioethics Committee of the Medical University of Bialystok (Approval No. RI-002/647/2019; APK.002.39.2021). All the analyses included in the study were carried out within a similar timeframe (July–August 2020) to ensure that the results obtained were reliable and valid. All the study participants were fully informed about the study procedures and were made aware of their right to withdraw from the study at any stage. Prior to the commencement of the study, they provided informed and written consent to participate.

### 4.1. Criteria for Participant Qualification for the Study

In the first stage of the study, 113 individuals (76 women and 37 men) aged 20 to 55 years were enrolled based on their medical history. The inclusion criterion for the study group was the presence of primary obesity (BMI ≥ 30 kg/m^2^). Individuals with normal body weight (BMI 18.5–24.9 kg/m^2^) were assigned to the control group. Exclusion criteria included secondary obesity, all types of diabetes, endocrine disorders, acute coronary syndrome, pharmacological or surgical treatment of obesity, chronic inflammatory diseases, malignant tumors, antiretroviral therapy, steroid therapy, pacemaker implantation, pregnancy, and lactation, as well as the use of hormonal contraception or hormone replacement therapy. For women, analyses were conducted during the follicular phase of the menstrual cycle (days 8–11 of the cycle).

The participants were divided into two groups, the study group and the control group, based on the criteria of body mass index (BMI) and total body fat content. For this purpose, anthropometric measurements, such as body weight and height, and waist and hip circumference, were taken, and body composition analysis was performed, to determine the content and distribution of fat tissue. WHR and VAT/SAT ratio were also calculated. Subsequently, all the individuals underwent biochemical tests, and the results were used to assess the actual presence of metabolic disorders and comorbidities.

The final group of participants consisted of 81 individuals (54 women and 27 men). The study group included 46 individuals (28 women and 18 men) with obesity classified as grade I and II (BMI = 30.0–39.9 kg/m^2^) and a total body fat content of >30% in women and >25% in men. The control group comprised 35 individuals (26 women and 9 men) with a normal body mass index (BMI = 18.5–24.9 kg/m^2^) and total body fat content ranging from 20 to 30% in women and 15 to 20% in men. The age range of the patients was 29–46 years.

In all the participants from the target groups, the serum concentrations of selected adipokines and cytokines (adiponectin, TNF-α, IL-6, NMP-2, NMP-9, resistin, IL-1β, IL-23) were measured, along with selected biochemical tests, including fasting bilirubin (mg/dL), urea (mg/dL), uric acid (mg/dL), creatinine (mg/dL), eGFR (mL/min), total cholesterol (mg/dL), HDL fraction (mg/dL), LDL (mg/dL), TG (mg/dL), AST (U/L), ALT (U/L), GGTP (U/L), alkaline phosphatase (IU/L), and CRP (mg/L). Additionally, an oral glucose tolerance test (OGTT) was performed to determine glucose levels (mg/dL) at one hour and two hours after consuming 75 g of glucose. Fasting insulin levels (µU/mL) were also assessed. Based on these parameters, the HOMA index was determined, and the result was normal for all the study participants.

### 4.2. Anthropometrics Parameters and Body Composition Analysis

The participants underwent measurements of their body weight and height using a scale (with an accuracy of 0.01 kg) and a stadiometer (with an accuracy of 0.5 cm) from RADWAG WPT 100/200 OW (Radom, Poland). Based on these measurements, their body mass index (BMI) was calculated. Additionally, their waist and hip circumferences were measured, and their waist-to-hip ratio (WHR) was calculated accordingly. Body composition analysis was subsequently performed using bioelectrical impedance analysis, and their cross-sectional area of abdominal fat was measured with a BioScan 920-2 device (Essex, UK). The patients were instructed to fast before the examination and to refrain from intense physical activity. The assessment was conducted in the morning hours.

Body composition analysis was conducted in a supine position (electrodes were placed on the right arm and right leg). The total body fat content was assessed (kg, %). The quantitative analysis of abdominal fat was conducted in a standing position (electrodes were placed at the level of the navel, along a horizontal line). The cross-sectional area of abdominal fat was determined, including visceral fat (cm^3^, %) and subcutaneous fat (cm^3^, %), and the visceral-to-subcutaneous fat ratio (VAT/SAT) was calculated. The results were processed using Maltron BioScan 920 v1.1 software [39].

### 4.3. Biochemical Analysis

All the biochemical tests were performed in the same laboratory, using standardized methods. The patients had 10 mL of blood drawn from the cubital vein. They were instructed to fast before the tests. Concentrations of total cholesterol, LDL cholesterol, HDL cholesterol, triglyceride, C-reactive protein, glucose, insulin, urea, creatinine, uric acid, and total bilirubin were assessed on ALINITY ci, Abbott.

The concentration of uric acid was assessed by the method with uricase, the concentration of urea by the method with urease, and the concentration of creatinine by the method with creatininase. The GFR index was determined based on the creatinine level. The concentration of glucose was assessed by the method with hexokinase, cholesterol by the method with cholesterol esterase, HDL by the method with accelerator selective detergent, LDL by the method with liquid selective detergent, triglyceride by the method with glycerol phosphate oxidase, total bilirubin by the method with diazonium salt, ALT and AST by the spectrophotometric method with NADH, GGTP by the method with L-gamma-glutamyl-3-carboxy-4-nitroanilide as a substrate, and ALP by the method with para-nitrophenyl phosphate, while serum C-reactive protein (CRP) concentrations were measured using the turbidimetric method, and insulin was measured using chemiluminescent microparticle immunoassay (CMIA).

### 4.4. Measurement of Adipokine and Cytokine Levels in Serum

Blood samples were taken from the patients and frozen at −80 °C. The serum levels of total adiponectin (Quantikine ELISA Human Total Adiponectin/Acrp30 Immunoassay, R&D Systems, Abingdon, UK), resistin (Quantikine ELISA Human Resistin Immunoassay, R&D Systems, Abingdon, UK), IL-6 (Quantikine ELISA Human IL-6 Immunoassay, R&D Systems, Abingdon, UK), TNFα (Quantikine ELISA Human TNFα Immunoassay, R&D Systems, Abingdon, UK), IL-1β (Quantikine ELISA Human IL-1β/IL-1F2 Immunoassay, R&D Systems, Abingdon, UK), IL-23 (Quantikine ELISA Human IL-23 Immunoassay, R&D Systems, Abingdon, UK), MMP-9 (Quantikine ELISA Human MMP-9 Immunoassay, R&D Systems, Abingdon, UK), and total MMP-2 (Quantikine ELISA Total MMP-2 Immunoassay, R&D Systems, Abingdon, UK) were assessed with enzyme-linked immunosorbent assay (ELISA), according to the manufacturer’s instructions.

### 4.5. Statistical Analysis

Data analysis was performed using parametric and non-parametric methods. In groups with adequate sample sizes, the Shapiro–Wilk test was used to assess the normality of the variable distributions. Comparisons between the two groups with normally distributed variables were conducted using Student’s *t*-test. The appropriate version of this test was selected based on the assessment of variance homogeneity. Due to the sample sizes or the lack of normal distribution, the Mann–Whitney U test was also used for comparing the two groups. The relationships between numerical variables were assessed using Spearman’s rank correlation test. Multivariate analysis was conducted using multiple regression models, with adipose tissue parameters as the modeled variables. Regression models were created for the following dependent variables: total body fat percentage, VAT (cm^2^), SAT (cm^2^), and the VAT/SAT ratio (separate models for women and men), with serum CRP levels and serum levels of adipokines and cytokines as independent variables. Statistical significance was set at *p* < 0.05.

## 5. Conclusions

The strongest predictor of impaired adipose tissue function in both women and men was interleukin 6 (IL-6). Potential markers in women could also include matrix metalloproteinase-2 (MMP-2) and resistin measured in blood serum. However, further studies are necessary to confirm their diagnostic value. In diagnosing carbohydrate metabolism disorders, the use of adiponectin, TNF-α, IL-6, resistin, IL-1β and IL-23 in women, and IL-23 in men should be considered. IL-6 may also show potential in the diagnosis of non-alcoholic fatty liver disease with metabolic dysfunction, as well as in complications related to the urinary system, regardless of sex. In women, matrix metalloproteinases, specifically MMP-2 and MMP-9, could become markers of lipid metabolism disorders. However, the results obtained in our study require confirmation through studies conducted on larger populations. Additionally, the findings suggest that sex is a significant factor influencing the regulation of inflammatory markers, likely due to specific hormonal dynamics or fat distribution in women.

## Figures and Tables

**Table 1:** Comparison of anthropometric and body composition parameters of women and men from the study and control groups.

Parameter	Women (n = 54)				*p* *	Men (n = 27)				*p* **
	Study Group (n = 28)		Control Group (n = 26)			Study Group (n = 18)		Control Group (n = 9)		
	Median	Q1–Q3	Median	Q1–Q3		Median	Q1–Q3	Median	Q1–Q3	
Age (years)	42.50	37.50–46.00	35.50	30.00–41.00	0.009 *	39.00	34.00–45.00	36.00	29.00–38.00	0.322
Height (cm)	166.75	163.00–170.25	165.50	161.00–170.00	0.687	183.50	175.00–190.00	181.00	174.00–182.00	0.212
Weight (kg)	95.75	90.35–99.35	63.00	58.00–66.50	<0.001 *	111.50	105.00–120.00	80.00	76.00–82.00	<0.001 **
BMI (kg/m^2^)	33.55	31.60–36.30	22.90	21.90–23.70	<0.001 *	33.25	32.00–34.00	24.70	24.30–24.92	<0.001 **
Waist circumference (cm)	105.00	100.50–110.75	80.50	78.00–85.00	<0.001 *	112.50	111.00–116.00	87.00	85.00–94.00	<0.001 **
Hip circumference (cm)	120.00	115.00–122.50	97.00	94.00–101.00	<0.001 *	116.00	114.00–120.00	103.00	102.00–107.00	<0.001 **
WHR	0.89	0.84–0.94	0.85	0.79–0.87	0.002 *	0.97	0.95–0.99	0.86	0.83–0.88	<0.001 **
Body fat (kg)	42.18	34.57–45.42	16.70	13.85–20.06	<0.001 *	37.56	33.79–40.91	16.05	13.62–18.02	<0.001 **
Body fat (%)	44.63	39.72–46.87	28.20	23.19–29.77	<0.001 *	33.69	30.72–35.43	19.43	17.86–21.24	<0.001 **
VAT (cm^2^)	235.00	170.50–333.50	90.50	64.00–125.00	<0.001 *	299.00	220.00–350.00	129.00	104.00–214.00	0.017
SAT (cm^2^)	121.50	103.00–143.50	64.50	54.00–92.00	<0.001 *	138.50	109.00–152.00	68.00	63.00–74.00	<0.001 **
VAT/SAT ratio	2.03	1.45–3.01	1.26	0.94–1.66	0.002 *	2.19	1.51–2.93	1.56	1.41–3.31	0.527

Q1–Q3: 1st–3rd Quartile, BMI—body mass index, WHR—waist-to-hip ratio, VAT—visceral adipose tissue, SAT—subcutaneous adipose tissue. Statistical significance (*p* < 0.05), *p* *—statistical differences between women from study and control groups, *p* **—statistical differences between men from study and control groups.

**Table 2:** Comparison of serum concentration of selected pro-inflammatory cytokines and adipokines between women and men from the study and control groups.

Parameter	Women (n = 54)				*p* *	Men (n = 27)				*p* **
	Study Group (n = 28)		Control Group (n = 26)			Study Group (n = 18)		Control Group (n = 9)		
	Median	Q1–Q3	Median	Q1–Q3		Median	Q1–Q3	Median	Q1–Q3	
Adiponectin (ng/mL)	7447.10	5416.95–9898.15	9697.75	6110.80–14,032.70	0.331	4464.65	3221.20–7872.30	4453.80	3983.70–5580.90	0.860
TNF-alfa (pg/mL)	2.78	0.52–5.57	3.90	2.30–4.84	0.306	3.74	2.93–6.20	5.66	4.84–6.41	0.433
IL-6 (pg/mL)	1.76	1.38–2.90	0.62	0.30–1.24	0.000 *	1.73	1.24–2.79	1.01	0.26–2.01	0.035 **
MMP-2 (ng/mL)	324.72	264.15–419.56	404.90	308.68–538.04	0.030 *	372.46	281.16–480.16	414.42	295.38–476.38	0.940
MMP-9 (ng/mL)	704.85	643.00–1138.60	729.75	584.90–1083.80	0.911	942.90	637.70–1420.20	655.10	463.10–992.50	0.275
Resistin (ng/mL)	13.89	9.39–17.83	10.13	8.59–12.62	0.029 *	9.77	8.02–15.54	9.50	7.26–10.37	0.322
IL-1β (pg/mL)	0.38	0.00–0.69	0.60	0.20–1.22	0.073	0.37	0.17–1.27	0.32	0.09–0.63	0.631
IL-23 (pg/mL)	1.23	0.00–4.43	1.94	0.91–3.97	0.176	0.00	0.00–1.20	0.37	0.00–1.20	0.298

IL-6—interleukin-6, MMP-2—metalloproteinase-2, MMP-9—metalloproteinase-9, IL-1β—interleukin 1β, IL-23—interleukin 23. Statistical significance (*p* < 0.05), *p* *—statistical differences between women from study and control groups, *p* **—statistical differences between men from study and control groups.

**Table 3:** Correlations between the concentrations of cytokines/adipokines in the serum and selected anthropometric/body composition parameters of women from the study and control groups.

Women (n = 54)								
Parameter	Adiponectin (ng/mL)	TNF-α (pg/mL)	IL-6 (pg/mL)	MMP-2 (ng/mL)	MMP-9 (ng/mL)	Resistin (ng/mL)	IL-1β (pg/mL)	IL-23 (pg/mL)
BMI (kg/m^2^)	r = −0.264	r = −0.168	r = 0.651	r = −0.357	r = 0.081	r = 0.255	r = −0.239	r = −0.174
	*p* = 0.054	*p* = 0.226	*p* = 0.000 *	*p* = 0.008 *	*p* = 0.559	*p* = 0.063	*p* = 0.082	*p* = 0.208
Waist circumference (cm)	r = −0.2221	r = −0.198	r = 0.629	r = −0.382	r = 0.020	r = 0.234	r = −0.309	r = −0.187
	*p* = 0.109	*p* = 0.151	*p* = 0.000 *	*p* = 0.004 *	*p* = 0.886	*p* = 0.088	*p* = 0.023 *	*p* = 0.177
Hip circumference (cm)	r = −0.175	r = −0.082	r = 0.649	r = −0.359	r = 0.023	r = 0.402	r = −0.216	r = −0.143
	*p* = 0.204	*p* = 0.554	*p* = 0.000 *	*p* = 0.008 *	*p* = 0.867	*p* = 0.003 *	*p* = 0.117	*p* = 0.301
WHR	r = −0.155	r = −0.294	r = 0.263	r = −0.235	r = 0.035	r = −0.151	r = −0.225	r = −0.248
	*p* = 0.263	*p* = 0.031 *	*p* = 0.055	*p* = 0.087	*p* = 0.800	*p* = 0.277	*p* = 0.102	*p* = 0.071
Body fat (kg)	r = −0.264	r = −0.103	r = 0.687	r = −0.387	r = 0.095	r = 0.308	r = −0.259	r = −0.168
	*p* = 0.054	*p* = 0.459	*p* = 0.000 *	*p* = 0.004 *	*p* = 0.493	*p* = 0.023 *	*p* = 0.059	*p* = 0.225
Body fat (%)	r = −0.245	r = −0.126	r = 0.682	r = −0.366	r = 0.126	r = 0.239	r = −0.288	r = −0.178
	*p* = 0.074	*p* = 0.364	*p* = 0.000 *	*p* = 0.006 *	*p* = 0.362	*p* = 0.082	*p* = 0.035 *	*p* = 0.198
VAT (cm^2^)	r = −0.193	r = −0.086	r = 0.596	r = −0.219	r = 0.085	r = 0.192	r = −0.382	r = −0.204
	*p* = 0.162	*p* = 0.539	*p* = 0.000 *	*p* = 0.112	*p* = 0.542	*p* = 0.165	*p* = 0.004 *	*p* = 0.139
SAT (cm^2^)	r = −0.159	r = −0.168	r = 0.478	r = −0.356	r = 0.037	r = 0.131	r = −0.368	r = −0.006
	*p* = 0.251	*p* = 0.225	*p* = 0.000 *	*p* = 0.008 *	*p* = 0.793	*p* = 0.346	*p* = 0.006 *	*p* = 0.966
VAT/SAT ratio	r = −0.133	r = −0.012	r = 0.402	r = −0.030	r = 0.105	r = 0.107	r = −0.257	r = −0.192
	*p* = 0.337	*p* = 0.932	*p* = 0.003 *	*p* = 0.830	*p* = 0.452	*p* = 0.443	*p* = 0.061	*p* = 0.165

BMI—body mass index, WHR—waist-to-hip ratio, VAT—visceral adipose tissue, SAT—subcutaneous adipose tissue. IL-6—interleukin-6, MMP-2—metalloproteinase-2, MMP-9—metalloproteinase-9, IL-1β—interleukin 1β, IL-23—interleukin 23. *p* *—statistical significance (*p* < 0.05).

**Table 4:** Correlations between the concentrations of cytokines/adipokines in the serum and selected anthropometric/body composition parameters of men from the study and control groups.

Men (n = 27)								
Parameter	Adiponectin (ng/mL)	TNF-α (pg/mL)	IL-6 (pg/mL)	MMP-2 (ng/mL)	MMP-9 (ng/mL)	Resistin (ng/mL)	IL-1β (pg/mL)	IL-23 (pg/mL)
BMI (kg/m^2^)	r = 0.175	r = 0.082	r = 0.238	r = 0.101	r = 0.185	r = 0.096	r = 0.214	r = −0.231
	*p* = 0.384	*p* = 0.684	*p* = 0.233	*p* = 0.614	*p* = 0.355	*p* = 0.634	*p* = 0.283	*p* = 0.246
Waist circumference (cm)	r = 0.091	r = 0.222	r = 0.451	r = −0.021	r = 0.187	r = 0.085	r = 0.067	r = −0.208
	*p* = 0.650	*p* = 0.266	*p* = 0.018 *	*p* = 0.917	*p* = 0.351	*p* = 0.674	*p* = 0.739	*p* = 0.297
Hip circumference (cm)	r = −0.167	r = 0.139	r = 0.501	r = −0.111	r = 0.298	r = 0.295	r = 0.052	r = −0.254
	*p* = 0.406	*p* = 0.489	*p* = 0.008 *	*p* = 0.580	*p* = 0.130	*p* = 0.135	*p* = 0.796	*p* = 0.201
WHR	r = 0.227	r = 0.163	r = 0.443	r = 0.069	r = 0.164	r = 0.025	r = 0.134	r = −0.195
	*p* = 0.255	*p* = 0.418	*p* = 0.021 *	*p* = 0.731	*p* = 0.415	*p* = 0.901	*p* = 0.507	*p* = 0.330
Body fat (kg)	r = 0.082	r = 0.055	r = 0.252	r = −0.087	r = 0.208	r = 0.170	r = 0.081	r = −0.170
	*p* = 0.683	*p* = 0.787	*p* = 0.206	*p* = 0.665	*p* = 0.297	*p* = 0.397	*p* = 0.689	*p* = 0.397
Body fat (%)	r = 0.103	r = −0.037	r = 0.168	r = −0.062	r = 0.177	r = 0.190	r = 0.144	r = −0.093
	*p* = 0.611	*p* = 0.854	*p* = 0.401	*p* = 0.758	*p* = 0.377	*p* = 0.341	*p* = 0.473	*p* = 0.643
VAT (cm^2^)	r = 0.042	r = 0.369	r = 0.208	r = 0.028	r = 0.002	r = 0.142	r = 0.071	r = −0.290
	*p* = 0.836	*p* = 0.578	*p* = 0.297	*p* = 0.888	*p* = 0.990	*p* = 0.481	*p* = 0.724	*p* = 0.142
SAT (cm^2^)	r = 0.205	r = 0.067	r = 0.347	r = −0.136	r = 0.240	r = 0.070	r = 0.111	r = −0.205
	*p* = 0.305	*p* = 0.741	*p* = 0.076	*p* = 0.499	*p* = 0.228	*p* = 0.278	*p* = 0.580	*p* = 0.305
VAT/SAT ratio	r = −0.222	r = 0.262	r = −0.054	r = 0.184	r = −0.115	r = 0.022	r = 0.003	r = −0.292
	*p* = 0.266	*p* = 0.187	*p* = 0.790	*p* = 0.359	*p* = 0.568	*p* = 0.914	*p* = 0.988	*p* = 0.139

BMI—body mass index, WHR—waist-to-hip ratio, VAT—visceral adipose tissue, SAT—subcutaneous adipose tissue. IL-6—interleukin-6, MMP-2—metalloproteinase-2, MMP-9—metalloproteinase-9, IL-1β—interleukin 1β, IL-23—interleukin 23. *p* *—statistical significance (*p* < 0.05).

**Table 5:** Comparison of biochemical parameters of women and men from the study and control groups.

Parameter	Women (n = 54)				*p* *	Men (n = 27)				*p* **
	Study Group (n = 28)		Control Group (n = 26)			Study Group (n = 18)		Control Group (n = 9)		
	Median	Q1–Q3	Median	Q1–Q3		Median	Q1–Q3	Median	Q1–Q3	
Bilirubin-T (mg/dL)	0.66	0.48–1.00	0.81	0.66–1.17	0.144	0.77	0.67–1.04	0.65	0.55–0.83	0.426
Urea (mg/dL)	23.54	21.40–27.82	21.40	19.26–25.68	0.159	25.68	23.54–27.82	25.68	25.68–29.96	0.781
Creatine (mg/dL)	0.77	0.69–0.83	0.67	0.65–0.79	0.035 *	0.88	0.79–0.96	0.88	0.83–0.95	0.668
eGFR (ml/min)	88.50	82.50–106.00	105.00	93.00–112.00	0.018 *	103.00	90.00–116.00	110.00	90.00–114.00	0.860
Uric acid (mg/dL)	5.10	4.65–5.65	4.30	3.90–4.60	0.000 *	6.75	6.00–7.30	5.30	4.90–5.80	0.004 **
Cholesterol-T (mg/dL)	193.00	179.00–222.00	193.00	180.00–209.00	0.966	208.50	184.00–235.00	183.00	170.00–209.00	0.160
HDL-C (mg/dL)	55.00	47.00–60.00	62.50	59.00–83.00	0.000 *	43.50	37.00–48.00	46.00	42.00–51.00	0.275
LDL-C (mg/dL)	121.00	99.00–138.50	93.50	71.00–112.00	0.002 *	140.00	108.00–154.00	104.00	98.00–124.00	0.046 **
TG (mg/dL)	96.00	67.50–127.00	62.00	52.00–83.00	0.001 *	135.50	89.00–173.00	92.00	78.00–105.00	0.031 **
AST (U/L)	17.00	15.00–20.00	17.00	15.00–19.00	0.911	25.50	19.00–28.00	21.00	19.00–23.00	0.176
ALT (U/L)	19.00	15.00–22.00	14.00	13.00–18.00	0.012 *	34.00	25.00–50.00	22.00	18.00–28.00	0.017 **
GGTP (U/L)	20.50	15.50–30.50	15.00	11.00–18.00	0.002 *	47.50	28.00–82.00	21.00	17.00–24.00	0.000 **
ALP (IU/L)	58.00	51.50–69.50	48.00	41.00–58.00	0.005 *	59.50	45.00–64.00	55.00	48.00–60.00	0.820
CRP (mg/L)	2.35	1.15–5.10	1.00	1.00–1.10	0.000 *	1.80	1.30–3.30	1.70	1.00–4.70	0.860
Fasting glucose (mg/dL)	94.00	85.50–97.00	89.50	85.00–92.00	0.085	99.00	89.00–106.00	93.00	90.00–96.00	0.160
Glucose after 1 h (mg/dL)	126.50	114.50–157.50	124.50	84.00–145.00	0.259	151.00	112.00–184.00	113.00	86.00–134.00	0.076
Glucose after 2 h (mg/dL)	104.00	98.00–131.00	100.00	89.00–113.00	0.238	101.00	89.00–115.00	82.00	71.00–93.00	0.023 **
Fasting insulin (µU/mL)	9.70	7.95–13.65	5.80	4.50–7.30	0.000 *	13.05	9.30–15.50	7.20	6.30–8.10	0.000 **

Q1–Q3: 1st–3rd Quartile, T—total, eGFR—estimated glomerular filtration rate, HDL-C—high-density lipoproteins, LDL-C—low-density lipoproteins, TG—triglycerides, AST– aspartate aminotransferase, ALT—alanine transaminase, GGTP—gamma-glutamyl transferase, ALP—alkaline phosphatase, CRP—C-reactive protein. Statistical significance (*p* < 0.05), *p* *—statistical differences between women from study and control groups, *p* **—statistical differences between men from study and control groups.

**Table 6:** Correlations between CRP levels and adipose tissue parameters in women and men.

CRP (mg/L)		
	Women (n = 54)	Men (n = 27)
Body fat (%)	r = 0.662	r = −0.113
	*p* = 0.000 *	*p* = 0.575
VAT (cm^2^)	r = 0.529	r = 0.032
	*p* < 0.001 *	*p* = 0.873
SAT (cm^2^)	r = 0.419	r = 0.129
	*p* = 0.002 *	*p* = 0.520
VAT/SAT ratio	r = 0.351	r = 0.073
	*p* = 0.009 *	*p* = 0.719

VAT—visceral adipose tissue, SAT—subcutaneous adipose tissue; *p* *—statistical significance (*p* < 0.05).

**Table 7:** Selected multiple regression models for adipose tissue parameters in the group of women.

Variable		CRP (mg/L)	Adiponectin (ng/mL)	TNF-alfa (pg/mL)	IL-6 (pg/mL)	MMP-2 (ng/mL)	MMP-9 (ng/mL)	Resistin (ng/mL)	IL-1B (pg/mL)	IL-23 (pg/mL)	R^2^ [Adj. R^2^]
Model 1	β	1.664	−0.001	−0.140	2.665	−0.005	0.001	−0.047	−0.874	−0.315	
Dependent variable:	SE	0.475	0.001	0.302	0.709	0.008	0.003	0.213	1.306	1.306	
Body fat (%)	*p*	0.001 *	0.010 *	0.646	<0.001 *	0.575	0.992	0.824	0.507	0.343	
											0.585
											[0.500]
Model 2	β	17.245	−0.003	2.041	8.159	0.007	0.025	2.713	−20.499	−7.125	
Dependent variable:	SE	5.901	0.002	3.757	8.811	0.105	0.035	2.642	16.227	4.086	
VAT (cm^2^)	*p*	0.005 *	0.110	0.590	0.360	0.949	0.480	0.310	0.213	0.088	
											0.411
											[0.291]
Model 3	β	7.696	−0.001	−2.111	−1.079	−0.053	−0.001	0.508	−7.420	−1.306	
Dependent variable:	SE	2.450	0.001	1.560	3.658	0.044	0.015	1.097	6.737	1.700	
SAT (cm^2^)	*p*	0.003 *	0.524	0.183	0.769	0.236	0.955	0.645	0.277	0.446	
											0.354
											[0.221]
Model 4	β	0.117	−0.001	0.060	0.015	0.001	0.001	0.030	−0.147	−0.046	
Dependent variable:	SE	0.076	0.001	0.049	0.114	0.001	0.001	0.034	0.210	0.053	
VAT/SAT ratio	*p*	0.133	0.317	0.225	0.897	0.838	0.358	0.392	0.486	0.388	
											0.199
											[0.035]

VAT—visceral adipose tissue, SAT—subcutaneous adipose tissue. IL-6—interleukin-6, MMP-2—metalloproteinase-2, MMP-9—metalloproteinase-9, IL-1β—interleukin 1β, IL-23—interleukin 23. *p* *—statistical significance (*p* < 0.05).

**Table 8:** Selected multiple regression models for adipose tissue parameters in the group of men.

Variable		CRP (mg/L)	Adiponectin (ng/mL)	TNF-alfa (pg/mL)	IL-6 (pg/mL)	MMP-2 (ng/mL)	MMP-9 (ng/mL)	Resistin (ng/mL)	IL-1B (pg/mL)	IL-23 (pg/mL)	R^2^ [Adj. R^2^]
Model 1	β	−0.131	−0.001	0.017	0.726	−0.010	−0.001	0.293	4.753	0.012	
Dependent variable:	SE	0.197	0.001	0.061	3.466	0.024	0.008	0.527	5.674	0.262	
Body fat (%)	*p*	0.513	0.854	0.788	0.837	0.676	0.976	0.586	0.414	0.964	
											0.166
											[--]
Model 2	β	0.572	−0.002	0.352	−2.665	0.030	−0.036	5.132	21.917	1.089	
Dependent variable:	SE	2.287	0.010	0.713	40.283	0.280	0.094	6.124	65.941	3.043	
VAT (cm^2^)	*p*	0.805	0.866	0.627	0.948	0.916	0.709	0.414	0.744	0.725	
											0.133
											[--]
Model 3	β	−0.610	−0.001	−0.127	12.596	−0.047	0.010	0.509	15.676	−0.205	
Dependent variable:	SE	0.823	0.003	0.256	14.491	0.101	0.034	2.203	23.720	1.094	
SAT (cm^2^)	*p*	0.469	0.796	0.626	0.397	0.648	0.782	0.820	0.518	0.853	
											0.253
											[--]
Model 4	β	0.033	−0.001	0.006	−0.294	0.002	−0.001	0.036	−0.238	0.011	
Dependent variable:	SE	0.022	0.001	0.007	0.385	0.003	0.001	0.059	0.630	0.029	
VAT/SAT ratio	*p*	0.150	0.692	0.418	0.455	0.580	0.477	0.542	0.711	0.710	
											0.54
											[0.013]

VAT—visceral adipose tissue, SAT—subcutaneous adipose tissue. IL-6—interleukin-6, MMP-2—metalloproteinase-2, MMP-9—metalloproteinase-9, IL-1β—interleukin 1β, IL-23—interleukin 23.

**Table 9:** Correlations between the concentrations of cytokines/adipokines in the serum and selected biochemical parameters of women from the study and control groups.

Women (n = 54)								
Parameter	Adiponectin (ng/mL)	TNF-α (pg/mL)	IL-6 (pg/mL)	MMP-2 (ng/mL)	MMP-9 (ng/mL)	Resistin (ng/mL)	IL-1β (pg/mL)	IL-23 (pg/mL)
Bilirubin-T (mg/dL)	r = −0.060	r = 0.179	r = −0.140	r = −0.241	r = −0.014	r = 0.067	r = −0.084	r = −0.162
	*p* = 0.669	*p* = 0.195	*p* = 0.313	*p* = 0.079	*p* = 0.921	*p* = 0.628	*p* = 0.544	*p* = 0.242
Urea (mg/dL)	r = −0.176	r = 0.158	r = 0.165	r = −0.128	r = 0.014	r = −0.033	r = 0.093	r = −0.071
	*p* = 0.202	*p* = 0.255	*p* = 0.234	*p* = 0.356	*p* = 0.921	*p* = 0.813	*p* = 0.503	*p* = 0.608
Creatine (mg/dL)	r = 0.020	r = −0.036	r = 0.159	r = −0.139	r = −0.178	r = 0.188	r = 0.108	r = −0.142
	*p* = 0.884	*p* = 0.797	*p* = 0.252	*p* = 0.316	*p* = 0.200	*p* = 0.172	*p* = 0.438	*p* = 0.306
eGFR (ml/min)	r = 0.046	r = −0.089	r = −0.218	r = 0.157	r = 0.148	r = −0.207	r = −0.020	r = 0.171
	*p* = 0.744	*p* = 0.523	*p* = 0.114	*p* = 0.257	*p* = 0.284	*p* = 0.134	*p* = 0.887	*p* = 0.217
Uric acid (mg/dL)	r = 0.014	r = 0.002	r = 0.336	r = −0.139	r = 0.041	r = 0.123	r = −0.179	r = −0.171
	*p* = 0.923	*p* = 0.987	*p* = 0.013 *	*p* = 0.316	*p* = 0.766	*p* = 0.375	*p* = 0.194	*p* = 0.215
Cholesterol-T (mg/dL)	r = 0.003	r = −0.250	r = −0.043	r = 0.109	r = 0.277	r = −0.252	r = −0.085	r = −0.107
	*p* = 0.985	*p* = 0.068	*p* = 0.759	*p* = 0.434	*p* = 0.043 *	*p* = 0.066	*p* = 0.540	*p* = 0.440
HDL-C (mg/dL)	r = 0.387	r = −0.154	r = −0.286	r = 0.378	r = −0.029	r = −0.177	r = 0.204	r = 0.014
	*p* = 0.004 *	*p* = 0.266	*p* = 0.036 *	*p* = 0.005 *	*p* = 0.833	*p* = 0.202	*p* = 0.140	*p* = 0.920
LDL-C (mg/dL)	r = −0.171	r = −0.231	r = 0.210	r = −0.109	r = 0.271	r = −0.110	r = −0.285	r = −0.146
	*p* = 0.217	*p* = 0.093	*p* = 0.127	*p* = 0.431	*p* = 0.047 *	*p* = 0.429	*p* = 0.037 *	*p* = 0.294
TG (mg/dL)	r = −0.047	r = −0.130	r = 0.446	r = −0.096	r = 0.389	r = 0.123	r = −0.243	r = −0.124
	*p* = 0.734	*p* = 0.347	*p* = 0.001 *	*p* = 0.490	*p* = 0.004 *	*p* = 0.374	*p* = 0.076	*p* = 0.370
AST (U/L)	r = 0.030	r = −0.014	r = 0.011	r = 0.166	r = −0.085	r = 0.116	r = 0.222	r = −0.211
	*p* = 0.829	*p* = 0.921	*p* = 0.937	*p* = 0.231	*p* = 0.543	*p* = 0.404	*p* = 0.107	*p* = 0.125
ALT (U/L)	r = 0.153	r = −0.143	r = 0.255	r = 0.006	r = −0.107	r = 0.124	r = −0.014	r = −0.092
	*p* = 0.268	*p* = 0.303	*p* = 0.062	*p* = 0.963	*p* = 0.441	*p* = 0.373	*p* = 0.921	*p* = 0.510
GGTP (U/L)	r = 0.056	r = −0.023	r = 0.340	r = −0.213	r = −0.017	r = 0.118	r = −0.154	r = 0.055
	*p* = 0.687	*p* = 0.091	*p* = 0.012 *	*p* = 0.122	*p* = 0.902	*p* = 0.396	*p* = 0.266	*p* = 0.695
ALP (IU/L)	r = 0.089	r = −0.150	r = 0.347	r = −0.120	r = −0.055	r = −0.044	r = −0.032	r = −0.328
	*p* = 0.523	*p* = 0.279	*p* = 0.010 *	*p* = 0.389	*p* = 0.690	*p* = 0.750	*p* = 0.821	*p* = 0.015 *
CRP (mg/L)	r = 0.057	r = −0.326	r = 0.548	r = −0.070	r = 0.334	r = 0.089	r = −0.036	r = −0.315
	*p* = 0.682	*p* = 0.016 *	*p* = 0.000 *	*p* = 0.615	*p* = 0.014 *	*p* = 0.521	*p* = 0.796	*p* = 0.020 *
Fasting glucose (mg/dL)	r = −0.307	r = −0.164	r = 0.133	r = −0.036	r = −0.020	r = 0.213	r = 0.128	r = 0.356
	*p* = 0.024 *	*p* = 0.236	*p* = 0.338	*p* = 0.795	*p* = 0.886	*p* = 0.122	*p* = 0.356	*p* = 0.008 *
Glucose after 1 h (mg/dL)	r = −0.055	r = −0.296	r = 0.232	r = 0.005	r = −0.002	r = 0.028	r = 0.055	r = 0.113
	*p* = 0.693	*p* = 0.030 *	*p* = 0.091	*p* = 0.971	*p* = 0.986	*p* = 0.839	*p* = 0.695	*p* = 0.416
Glucose after 2 h (mg/dL)	r = 0.107	r = −0.252	r = 0.234	r = 0.207	r = 0.209	r = −0.195	r = 0.071	r = −0.032
	*p* = 0.441	*p* = 0.066	*p* = 0.088	*p* = 0.134	*p* = 0.129	*p* = 0.157	*p* = 0.612	*p* = 0.821
Fasting insulin (µU/mL)	r = −0.194	r = −0.075	r = 0.472	r = −0.214	r = −0.126	r = 0.176	r = −0.355	r = −0.020
	*p* = 0.160 *	*p* = 0.589	*p* = 0.000 *	*p* = 0.120	*p* = 0.365	*p* = 0.203 *	*p* = 0.008 *	*p* = 0.883

Q1–Q3: 1st–3rd Quartile, T—total, eGFR—estimated glomerular filtration rate, HDL-C—high-density lipoproteins, LDL-C—low-density lipoproteins, TG—triglycerides, AST—aspartate aminotransferase, ALT—alanine transaminase, GGTP—gamma-glutamyl transferase, ALP—alkaline phosphatase, CRP—C-reactive protein, *p* *—statistical significance (*p* < 0.05).

**Table 10:** Correlations between the concentrations of cytokines/adipokines in the serum and selected biochemical parameters of men from the study and control groups.

Men (n = 27)								
Parameter	Adiponectin (ng/mL)	TNF-α (pg/mL)	IL-6 (pg/mL)	MMP-2 (ng/mL)	MMP-9 (ng/mL)	Resistin (ng/mL)	IL-1β (pg/mL)	IL-23 (pg/mL)
Bilirubin-T (mg/dL)	r = 0.178	r = 0.094	r = −0.070	r = −0.171	r = 0.405	r = 0.148	r = 0.142	r = 0.063
	*p* = 0.383	*p* = 0.647	*p* = 0.732	*p* = 0.404	*p* = 0.040 *	*p* = 0.471	*p* = 0.489	*p* = 0.762
Urea (mg/dL)	r = −0.110	r = 0.010	r = −0.386	r = 0.182	r = −0.337	r = −0.248	r = −0.214	r = −0.166
	*p* = 0.586	*p* = 0.960	*p* = 0.047 *	*p* = 0.363	*p* = 0.085	*p* = 0.213	*p* = 0.284	*p* = 0.409
Creatine (mg/dL)	r = −0.061	r = 0.057	r = 0.251	r = −0.314	r = 0.067	r = 0.185	r = −0.117	r = 0.028
	*p* = 0.761	*p* = 0.777	*p* = 0.207	*p* = 0.111	*p* = 0.740	*p* = 0.357	*p* = 0.563	*p* = 0.889
eGFR (ml/min)	r = −0.072	r = −0.168	r = −0.358	r = 0.274	r = −0.008	r = −0.217	r = −0.006	r = −0.084
	*p* = 0.722	*p* = 0.401	*p* = 0.067	*p* = 0.166	*p* = 0.969	*p* = 0.277	*p* = 0.978	*p* = 0.676
Uric acid (mg/dL)	r = −0.137	r = 0.041	r = 0.285	r = −0.195	r = 0.177	r = 0.068	r = 0.006	r = −0.141
	*p* = 0.494	*p* = 0.840	*p* = 0.150	*p* = 0.331	*p* = 0.378	*p* = 0.736	*p* = 0.978	*p* = 0.483
Cholesterol-T (mg/dL)	r = 0.129	r = 0.084	r = 0.101	r = −0.290	r = −0.047	r = −0.125	r = −0.180	r = −0.093
	*p* = 0.522	*p* = 0.677	*p* = 0.617	*p* = 0.142	*p* = 0.815	*p* = 0.534	*p* = 0.369	*p* = 0.643
HDL-C (mg/dL)	r = 0.205	r = 0.133	r = −0.010	r = −0.158	r = −0.135	r = 0.017	r = 0.185	r = 0.256
	*p* = 0.305	*p* = 0.509	*p* = 0.960	*p* = 0.431	*p* = 0.503	*p* = 0.931	*p* = 0.355	*p* = 0.197
LDL-C (mg/dL)	r = 0.308	r = 0.019	r = 0.110	r = −0.058	r = 0.144	r = −0.002	r = 0.020	r = −0.158
	*p* = 0.118	*p* = 0.924	*p* = 0.584	*p* = 0.773	*p* = 0.473	*p* = 0.993	*p* = 0.919	*p* = 0.433
TG (mg/dL)	r = −0.270	r = −0.157	r = 0.104	r = −0.288	r = 0.025	r = −0.064	r = −0.347	r = −0.082
	*p* = 0.173	*p* = 0.435	*p* = 0.605	*p* = 0.145	*p* = 0.900	*p* = 0.753	*p* = 0.076	*p* = 0.684
AST (U/L)	r = −0.047	r = −0.015	r = 0.033	r = 0.043	r = −0.149	r = −0.251	r = 0.201	r = −0.095
	*p* = 0.815	*p* = 0.940	*p* = 0.870	*p* = 0.831	*p* = 0.459	*p* = 0.206	*p* = 0.315	*p* = 0.637
ALT (U/L)	r = −0.048	r = −0.396	r = 0.050	r = 0.018	r = 0.072	r = −0.144	r = 0.068	r = −0.162
	*p* = 0.813	*p* = 0.041 *	*p* = 0.805	*p* = 0.929	*p* = 0.722	*p* = 0.744	*p* = 0.738	*p* = 0.420
GGTP (U/L)	r = −0.141	r = −0.153	r = 0.378	r = −0.226	r = 0.020	r = −0.019	r = −0.005	r = −0.021
	*p* = 0.484	*p* = 0.447	*p* = 0.052	*p* = 0.258	*p* = 0.920	*p* = 0.926	*p* = 0.979	*p* = 0.917
ALP (IU/L)	r = −0.018	r = 0.031	r = 0.039	r = −0.155	r = 0.125	r = −0.259	r = −0.204	r = 0.008
	*p* = 0.929	*p* = 0.878	*p* = 0.846	*p* = 0.439	*p* = 0.535	*p* = 0.192	*p* = 0.308	*p* = 0.969
CRP (mg/L)	r = −0.362	r = 0.024	r = 0.294	r = −0.172	r = 0.207	r = 0.052	r = 0.074	r = −0.358
	*p* = 0.064	*p* = 0.904	*p* = 0.137	*p* = 0.390	*p* = 0.300	*p* = 0.798	*p* = 0.715	*p* = 0.067
Fasting glucose (mg/dL)	r = 0.159	r = 0.222	r = 0.184	r = 0.136	r = −0.073	r = −0.128	r = 0.079	r = −0.112
	*p* = 0.429	*p* = 0.265	*p* = 0.359	*p* = 0.500	*p* = 0.716	*p* = 0.525	*p* = 0.695	*p* = 0.579
Glucose after 1 h (mg/dL)	r = −0.095	r = −0.013	r = 0.218	r = 0.008	r = 0.282	r = −0.044	r = −0.176	r = −0.038
	*p* = 0.639	*p* = 0.948	*p* = 0.275	*p* = 0.970	*p* = 0.153	*p* = 0.828	*p* = 0.381	*p* = 0.851
Glucose after 2 h (mg/dL)	r = −0.095	r = −0.165	r = −0.182	r = 0.087	r = 0.162	r = 0.057	r = 0.009	r = −0.381
	*p* = 0.638	*p* = 0.412	*p* = 0.363	*p* = 0.667	*p* = 0.420	*p* = 0.777	*p* = 0.965	*p* = 0.050 *
Fasting insulin (µU/mL)	r = −0.112	r = −0.158	r = 0.215	r = 0.201	r = 0.072	r = −0.195	r = −0.017	r = −0.267
	*p* = 0.579	*p* = 0.432	*p* = 0.282	*p* = 0.316	*p* = 0.720	*p* = 0.329	*p* = 0.931	*p* = 0.179

Q1–Q3: 1st–3rd Quartile, T—total, eGFR—estimated glomerular filtration rate, HDL-C—high-density lipoproteins, LDL-C—low-density lipoproteins, TG—triglycerides, AST—aspartate aminotransferase, ALT—alanine transaminase, GGTP—gamma-glutamyl transferase, ALP—alkaline phosphatase, CRP—C-reactive protein, *p* *—statistical significance (*p* < 0.05).

## Data Availability

The data presented in this study are available on request from the corresponding author. The data are not publicly available due to privacy and ethical reasons.
